# Unraveling varying spatiotemporal patterns of Dengue Fever and associated exposure-response relationships with environmental variables in three Southeast Asian countries before and during COVID-19

**DOI:** 10.1371/journal.pntd.0012096

**Published:** 2025-04-28

**Authors:** Wei Luo, Zhihao Liu, Yiding Ran, Mengqi Li, Yuxuan Zhou, Weitao Hou, Shengjie Lai, Sabrina L. Li, Ling Yin

**Affiliations:** 1 GeoSpatialX Lab, Department of Geography, National University of Singapore, Singapore, Singapore; 2 Saw Swee Hock School of Public Health, National University of Singapore, Singapore, Singapore; 3 Department of Geography, The University of Hong Kong, Hong Kong, China; 4 Department of Geography, University of Zurich, Zurich, Switzerland; 5 Department of Architecture and Civil Engineering, City University of Hong Kong, Hong Kong Special Administrative Region, China; 6 School of Design and the Built Environment, Curtin University, Perth, Western Australia, Australia; 7 WorldPop, School of Geography and Environmental Science, University of Southampton, Southampton, United Kingdom; 8 School of Geography, University of Nottingham, Nottingham, United Kingdom; 9 Shenzhen Institute of Advanced Technology, Chinese Academy of Sciences, Shenzhen, China; Australian Red Cross Lifeblood, AUSTRALIA

## Abstract

The enforcement of COVID-19 interventions by diverse governmental bodies, coupled with the indirect impact of COVID-19 on short-term environmental changes (e.g., plant shutdowns lead to lower greenhouse gas emissions), influences the Dengue Fever (DF) vector. This provides a unique opportunity to investigate the indirect impact of COVID-19 on DF transmission and generate insights for targeted prevention measures. We aim to compare DF transmission patterns and the exposure-response relationship of environmental variables and DF incidence in the pre- and during-COVID-19 to identify variations and assess the indirect impact of COVID-19 on DF transmission. We initially visualized the overall trend of DF transmission from 2017-2022, then conducted two quantitative analyses to compare DF transmission pre-COVID-19 (2017–2019) and during-COVID-19 (2020–2022). These analyses included time series analysis to assess DF seasonality, and a Distributed Lag Non-linear Model (DLNM) to quantify the exposure-response relationship between environmental variables and DF incidence. We observed a notable surge in Singapore during-COVID-19, particularly from May to August in 2020 and 2022, with cases multiplying several times compared to pre-COVID-19. All subregions in Thailand exhibited remarkable synchrony with a similar annual trend except 2021. Cyclic patterns remained generally consistent, but seasonal variability in Singapore has become increasingly pronounced. Monthly DF incidence in three countries varied significantly. Exposure-response relationships of DF and environmental variables show varying degrees of change, notably in Northern Thailand, where the peak relative risk for the maximum temperature-DF relationship rose from about 3–17, and the max RR of overall cumulative association 0–3 months of relative humidity increased from around 4–40. Our study is the first to compare DF transmission patterns and their relationship with environmental variables before and during COVID-19, demonstrating that the pandemic has affected DF transmission and altered the exposure-response relationship at both national and regional levels.

## Author summary

Dengue Fever (DF) is a typical tropical disease transmitted via mosquito bites. COVID-19 lockdowns have altered human-mosquito contact patterns that impacted DF transmission. Additionally, lockdowns caused short-term environmental changes that affected DF vector breeding. In fact, during the COVID-19 period, the normal prevention and treatment of DF in many DF-endemic countries was negatively affected due to the sweep of COVID-19, such as strained allocation of medical resources and misreporting of cases. Therefore, this offers a unique chance to study the indirect impact of COVID-19 on DF transmission, guiding targeted and reasonable prevention measures. We used a series of analytical approaches including time series analysis, space-time scan statistics, and distributed lag non-linear model to compare the differences in DF transmission patterns and its exposure-response relationships with four environmental variables (average monthly precipitation, average monthly relative humidity, monthly maximum temperature, and monthly minimum temperature) before and during COVID-19 in three Southeast Asian (SEA) countries: Malaysia, Singapore and Thailand at the province scale. We found that the DF transmission pattern and its relationship with the environmental variables changed differently. For instance, seasonality and infections heightened in Singapore during COVID-19 and peak relative risk between max temperature and DF has rose significantly in Northern Thailand.

## 1. Introduction

Dengue Fever (DF), caused by four virus serotypes (DENV 1–4) and transmitted by mosquitoes, manifests as flu-like symptoms, including fever and headache [1]. The ecology of DF is influenced by environmental factors affecting vector dynamics, virus development, and mosquito-human interactions [2]. It has become a growing threat due to the increasing number of reported cases and deaths worldwide. The World Health Organization (WHO) reports that cases rose from 505,430 in 2000 to 5.2 million in 2019, and deaths more than quadrupled from 960 in 2000–4,032 in 2015 [1], significantly burdening public health and socio-economic development in many countries [3]. The COVID-19 pandemic’s onset in 2020 introduced significant societal disruptions, complicating DF transmission dynamics [4,5]. To curb the spread of the virus, countries introduced diverse public health measures such as quarantine, isolation, social distancing, and mask-wearing [6,7]. These interventions, coupled with the public’s fear of contracting COVID-19, prompted many individuals to remain indoors, thereby altering human mobility and contact patterns [8,9].Close human-mosquito contact is crucial for DF transmission [10–13], but it is decreased in 2020 due to the COVID-19-related interventions, such as in Americas and SEA [1,4]. However, many scholars believe otherwise that the pandemic actually deteriorated DF transmission as it strained healthcare systems and limited resources for DF prevention and vector control [4,14,15]. The declining trend shown by the data is merely the result of under-reporting of DF cases during the pandemic [14].

SEA is an endemic region for DF [16–18] approximately with 2.9 million DF episodes and 5,906 DF-related deaths occurring annually. The region suffers from an economic burden of roughly $950 million from DF [19]. During the COVID-19 pandemic, the number of DF cases in the region decreased, but some countries like Singapore experienced an increase in cases [4,20]. These inconsistent patterns at the regional and national levels indicate a complex situation that requires further investigation.

Researchers are increasingly focusing on the complex, indirect impact of COVID-19 and related interventions on DF transmission in SEA, where both COVID-19 and DF pose significant public health burdens. Ong and Mohd [21] utilized a Seasonal Autoregressive Integrated Moving Average (SARIMA) model to investigate the implications of lockdown on DF transmission in Malaysia,uncovering a potential diffusive effect of the DF vector that accelerated risk of DF infectious. Additionally, Chen et al. [4] quantified the effects of COVID-19-related disruptions on DF transmission in SEA and Latin America, revealing a strong relationship between interventions and reduced DF risk. Moreover, COVID-19 interventions have indirectly caused short-term environmental changes [22],indicating that DF incidence and its relationship with environmental variables may be influenced by these indirect effects. The exposure-response relationship between environmental variables and the DF incidence has received widespread attention [23]. Zhiwei Xu et al. [24] conducted a zonal investigation into the lagged effects of mean temperature and relative humidity on DF incidence in Thailand by using DLNM from 1999 to 2014, revealing significant relationship between the two factors but noteworthy spatial differences.

The recent emergence of the pandemic has led to decreased government attention to DF, with instances of misjudgment and under-reporting resulting in limited availability of accurate DF case data [25,26]. Many of the existing studies only focus on one country or province. Although some studies have addressed the issue at a regional level, they often overlook analyses at a finer spatial scale (e.g., first-level administrative), as observed in Chen et al. [4]. This limitation neglects potential regional synchrony and spatial heterogeneity, overestimates the significance of observations, and impedes the generation of insights that can be applied to a large area with a finer spatial scale. Furthermore, the majority of current studies concentrate solely on DF incidences during the pandemic as a means of comparison to investigate the indirect impact of COVID-19 [20,27]. However, DF incidences can be cyclical [28] so restricting the study period solely to the pandemic duration has the potential to introduce bias into the results. As highlighted by Chen et al. [4], 2019 experienced the most significant global outbreak of DF in history, complicating the observation and attribution of COVID-19’s effects. DF transmission varies annually due to both seasonal and cyclic patterns. Given the indirect impact of the pandemic on DF transmission during the past three years, it is imperative to consider all three years comprehensively. However, most existing studies have focused solely on either 2020 or 2021 without taking a holistic approach. In addition, existing research predominantly employs quantitative analysis methods to investigate the influence of environmental variables on DF, yet overlooks the indirect impacts of COVID-19 lockdown measures on short-term environmental change may result in shifts in the relationship between environmental variables and DF during the pandemic [22,24,29–35].

Therefore, we aim to enhance understanding of the indirect impact of the pandemic on DF transmission. To achieve this, we compared spatio-temporal patterns of DF transmission and the exposure-response relationship between environmental variables and DF cases in SEA, with a specific focus on Thailand, Malaysia, and Singapore, and we categorized DF cases and related environmental data into pre-COVID-19 (January 2017 to December 2019) and during-COVID-19 (January 2020 to December 2022). By doing so, we gained a comprehensive comparison and understanding of the two periods of the overall transmission trend and seasonality of DF cases in the region using time-series analysis. DLNM was then used to analyze the different relationship of environmental variables with DF incidences in the two periods taking the impact of lockdown measures on human mobility into consideration.

Our study provides new insights into how DF outbreaks have evolved in these three SEA countries pre and during COVID-19 and the indirect impact of COVID-19 on DF to generate a deeper understanding of DF infection. By informing policies that improve the efficiency of preventing and monitoring DF cases in the during-COVID recovery era, we hope to contribute to the fight against this disease.

## 2. Methods

### 2.1. Ethics statement

All the data is open accessed. DF data are mainly provided by webpage of health-related department in each countries, partly from a open dataset of a previous study [4]. Environmental variables are obtained from Google Earth Engine. Non-Pharmaceutical Interventions (NPI) data were obtained from the Oxford COVID-19 Government Response Tracker (OxCGRT) [36]. Therefore, it does not require the permission of any institution, individual or other unit.

### 2.2. Data

Our study focuses on the DF situation in SEA by comparing pre-COVID-19(2017–2019) and during-COVID-19(2020–2022). We collected DF surveillance data and environmental data for three countries at the provincial level from 2017 to 2022, including Singapore, Malaysia, and Thailan. With the exception of Singapore, which only provides data at the country level, provinces represent the first administrative tier. As Singapore’s population and land areas are similar to some provinces in other countries, Singapore’s country- level data can be compared with Malaysia’s and Thailand’s province-level data [37,38].

DF data for Malaysia were provided by the Ministry of Health, Malaysia [39]. DF data for Thailand were provided by the Bureau of Epidemiology, Thailand [40] and the data for Singapore were provided by the Ministry of Health, Singapore [41]. For Thailand, monthly DF data is available whereas for Malaysia and Singapore, only weekly data is provided. Of the 313 weeks of data for Malaysia, 31 weeks were missing. Missing values were estimated using linear interpolation [42]. For standardization, DF data for both Malaysia and Singapore are aggregated to obtain monthly data, for weeks spanning multiple months, we distributed the cases evenly across each day. The total population data for each country is sourced from the World Bank Group [43]. Moreover, employing Google Earth Engine, we gathered environmental variables in the study region spanning from 2017 to 2022. We selected four environmental variables that have been confirmed to be associated with DF transmission [2,44,45], including average monthly precipitation, average monthly relative humidity, monthly maximum temperature, and monthly minimum temperature. Since the implementation of restrictive measures across different regions has led to alterations in both human mobility and consequently in human-mosquito contact patterns, we also collected NPI data (OxCGRT project’s overall stringency index) from 2020 to 2022 that serves as a fixed variable in environmental-related analyses, facilitating the simulation of more authentic human mobility patterns during the COVID-19 period. This index, derived from various restrictions related to public health and social measures, and the value ranges from 0 to 100, with higher values representing stronger human mobility restrictions [36].

### 2.3. Time series analysis

We perform temporal variation analysis on the DF data. Line graphs were used to visualize overall monthly trends in DF cases across the three countries, displayed both as absolute case numbers and as proportions of the total population. Additionally, heatmaps were utilized to depict the DF infection trends across different subregions of Thailand, as well as in Singapore and Malaysia, to further explore regional synchronicity and patterns of change. Box plots were employed to provide a more detailed representation of yearly variations and seasonality. Due to the high seasonality nature of DF [46], we apply seasonal and trend decomposition using loess (STL) to decompose the time series of each country. STL separates time series data into three components: trend (representing long-term and low-frequency variations), seasonal (capturing variations within the same period), and random or remainder (accounting for residual variations after extracting the trend and seasonal components) [47]. Its advantages lie in its simplicity, robustness of results, and effectiveness in data visualization. The equation can be described as follows.


$$\begin{array}{c} y_{t}=T_{t}+S_{t}+R_{t} \end{array}$$ (1)

In this context, yt represents the response variable (i.e., case counts) at time t, while Tt, St, and Rt correspond to the trend, seasonal, and remainder components at the same time point. In addition, we also conducted retrospective space-time scan statistics to delineate the spatiotemporal patterns of DF transmission before and during the COVID-19 (S1 Text).

### 2.4. Exposure-response relationship between environmental variables and DF incidence

DLNM offers robust methodologies for exploring the exposure-response relationship between variables from distinct domains. Particularly within the realm of environmental variables, its reliability surpasses that of traditional time series analyses [48,49]. DLNM proves to be an invaluable tool for investigating the lagged relationship of DF incidence with environmental variables [22,24,29–33,35]. Leveraging the ‘dlnm’ package in R Studio, we conducted comprehensive analyses to compare exposure-response relationship between environmental variables and DF incidence both pre-COVID and during-COVID periods. This approach aims to deepen the understanding of how environmental factors influence DF incidence.

In this study, the analysis is divided into two distinct phases. Throughout these phases, we employed four key environmental variables (average monthly precipitation, average monthly relative humidity, monthly maximum temperature, and monthly minimum temperature) to illustrate and elucidate the processes under investigation. In the first phase, individual models were constructed for each sub-regions, aiming to explore the relationship between environmental variables and DF incidences. A cross-basis was employed for this purpose, utilizing a B-spline with two degrees of freedom (dfs) for the environmental variable space. The specific spline related to environmental variables was positioned at the point representing the lowest risk of DF. Due to the varying stringency of government responses to COVID-19 across different regions, human mobility was reduced to differing extents, resulting in varied changes in human-mosquito contact patterns. In light of the decreased human mobility associated with COVID-19 lockdowns, our analysis of the environmental-dengue fever (DF) exposure-response relationship incorporates the Oxford COVID-19 Government Response Tracker (OxCGRT) overall stringency index as a fixed variable. This approach enables a more accurate examination of the relationship between environmental factors and DF cases. To accommodate seasonal variations and long-term trends within the model, both month and year were integrated as dummy variables. Moving on to the second phase, a multivariate meta-analysis approach was embraced to synthesize the relationship between environmental variables and DF incidence across diverse areas. This process involved identifying specific associations, considering various lag times for the regions studied. In the analysis of the overall cumulative association, we set the lag period to three months. This decision was based on the Generalized Cross-Validation (GCV) score and supported by previous studies, which indicate that the impact of various environmental factors on DF is most pronounced within three months [35,50,51]. The initial phase of analysis was governed by the following mathematical model:


$$\begin{array}{c} Y_{t}\sim Poisson\left(\mu_{t}\right) \end{array}$$ (2)

$$\begin{array}{c} Log\left(\mu_{t}\right)=\alpha +\beta T_{t,1}+SI+\eta 1Month+\eta 2Year \end{array}$$ (3)

In the provided equation, where t represents the month, Yt signifies the number of DF cases in a given month, α denotes the model intercept, and β is the vector of coefficients for Tt,1 and the lag month. Tt,1 represents the matrix derived through DLNM for each type of environmental factor. SI represents Stringency Index.

**Fig 1:**
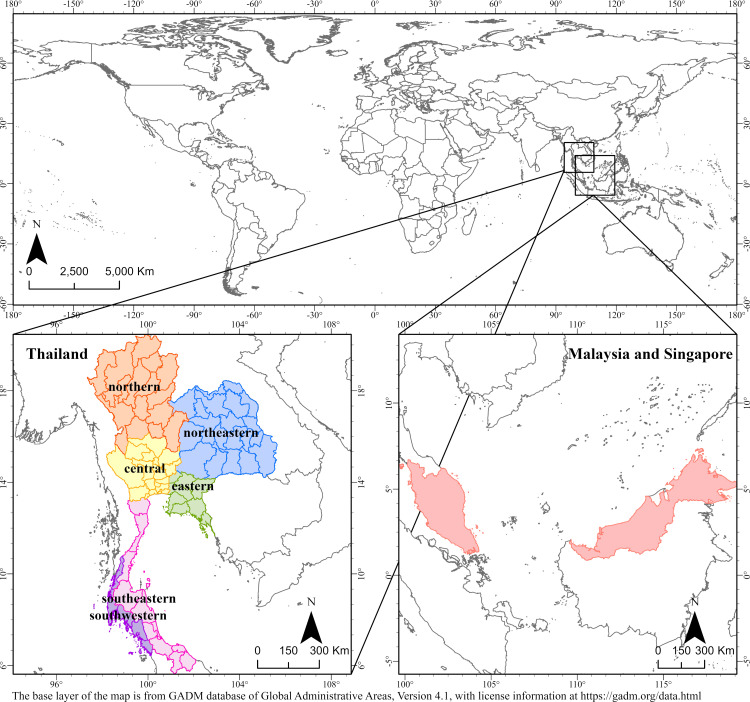
Study areas in the exposure-response relationship between DF incidence and environmental variables. (The base layer of the map is from GADM database of Global Administrative Areas, Version 4.1, with license information at https://gadm.org/data.html).

For analyzing the exposure-response relationship between DF incidence and environmental variables, given the substantial geographical expanse of Thailand and the comparatively smaller size of Singapore, we partitioned Thailand into six distinct study areas based on meteorological classification by the Thai Meteorological Department [52].While Malaysia and Singapore constituted a singular study area. This is done to take into account geographic scale and meteorological condition similarities [24,53,54], facilitating the understanding of the relationships between DF and different environmental conditions and providing more locally tailored recommendations for disease control. The delineation of these study areas is visually depicted in Fig 1.

## 3. Results

### 3.1. Overall trends

**Fig 2:**
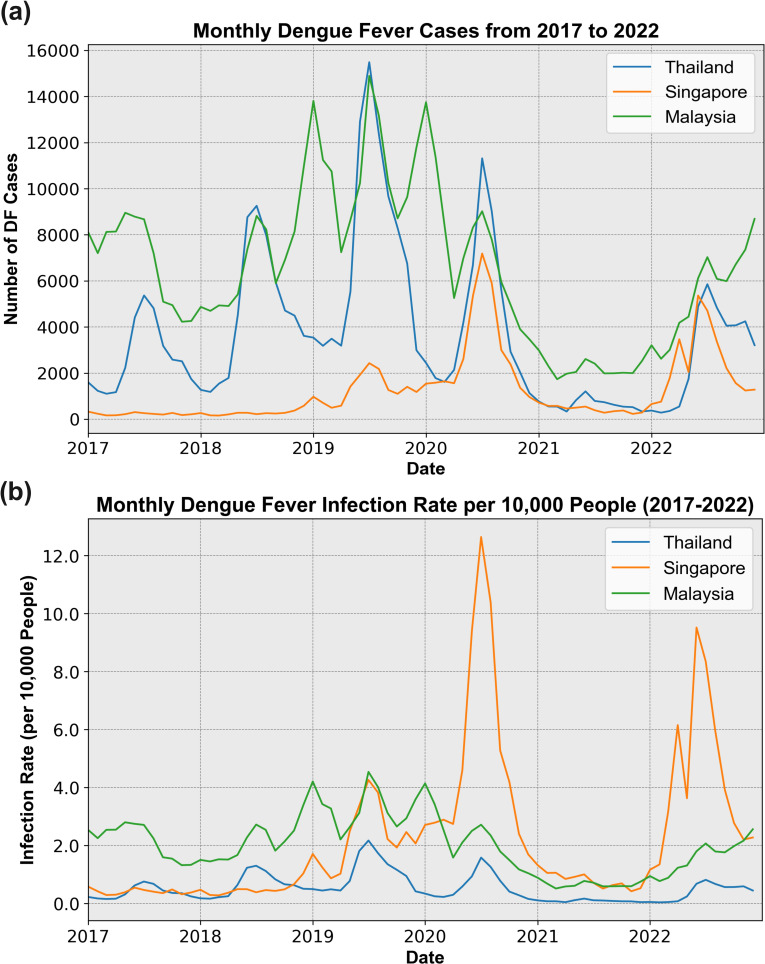
Monthly DF trend in three countries. a. DF cases. b. DF infection rate.

The DF infection trend exhibited varying degrees of change across the three countries during the COVID-19, compared with the 2017–2019 period. As illustrated in Fig 2a, during the COVID-19, DF cases peaked in 2020 in all three countries, with Thailand reaching approximately 11,000 cases. Mid-2022 saw another peak, with Malaysia recording a maximum of about 7,900 cases. Subsequently, Malaysia experienced a slight decline before continuing to rise, reaching a new peak of approximately 8,600 cases by the end of 2022. Notably, there was an overall decline in DF cases, except in Singapore, compared with the pre-COVID-19 period. Over the six-year period from 2017 to 2022, the trends in DF infections across the three countries were strikingly similar, with peaks and declines occurring almost synchronously during the COVID-19 period. Fig 2b illustrates the proportion of DF infections relative to the total population in the three countries. Remarkably, Singapore experienced significant infection peaks in mid-2020 and again at the beginning and middle of 2022, with the highest infection rate observed in mid-2020, reaching approximately 13 cases per 10,000 people. In contrast, infection rates in Malaysia and Thailand remained relatively stable, with a slight decrease compared to pre-COVID-19 period. Malaysia showed a pronounced increase in infection rates in mid-2020, followed by a continued rise throughout 2022.

**Fig 3:**
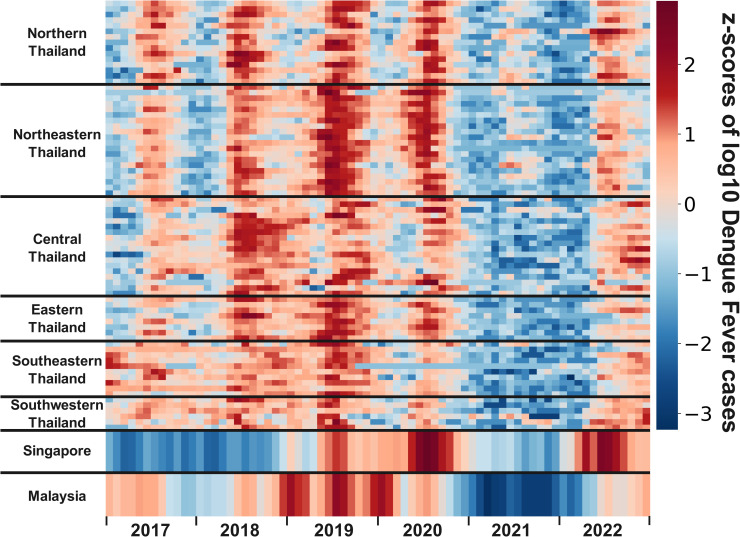
Monthly DF trend in all subregions.

From a subregional perspective (Fig 3), Generally, all sub-regions experience an increase in DF infections during the spring and summer each year. However, in 2021, DF cases sharply declined across the three countries during these seasons, though the northern and northeastern regions of Thailand experienced a slight increase. Furthermore, the pattern of DF transmission exhibits interannual seasonality. Upon closer observation, the peak incidence of DF in Northern and Northeastern Thailand occurs earlier and concludes sooner each year compared to other subregions of the country. The variation trends in DF cases for Singapore and Malaysia from 2017 to 2022 are similar to those of Thailand, with the exception of Singapore’s data from 2017 to 2018.

### 3.2. Time series analysis for seasonal variations

**Fig 4:**
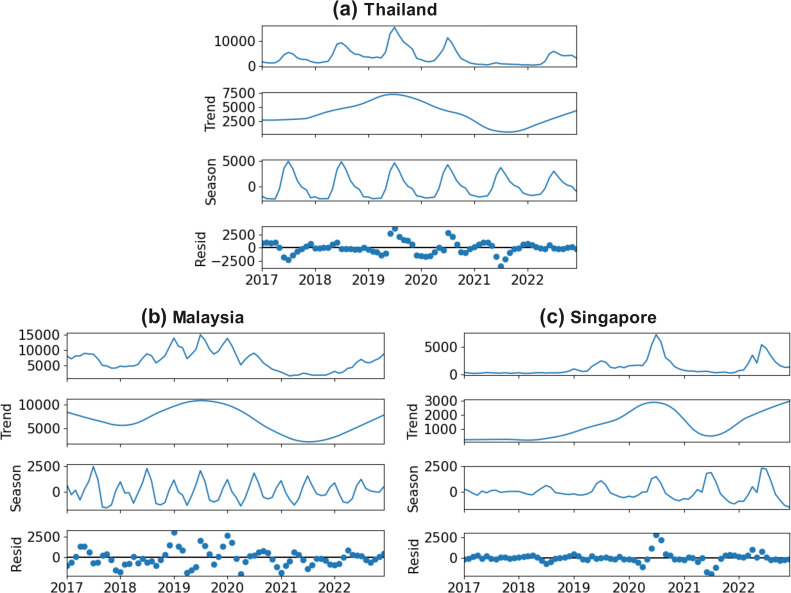
Seasonal Patterns of DF Incidences. a. Seasonal patterns in Thailand. b. Seasonal patterns in Malaysia. c Seasonal patterns in Singapore.

With STL, we decompose the DF incidences into yearly trends, seasonal variations, and residue shown in Fig 4. The cyclic patterns observed in the trend components coincide with those shown in Fig 3 whereas the seasonal components highlight the seasonality of DF cases. In general, the major peak of DF cases is around the middle of the year in all three countries, while the number of cases was low at the beginning and end of the year. Interestingly, the seasonal component in Thailand exhibits a gradual and subtle weakening trend over the years (Fig 4a), whereas in Singapore, the seasonal fluctuations have progressively intensified (Fig 4c). Malaysia exhibits a distinctive pattern in its peak values, with two notable troughs occurring at the beginning and end of the year (Fig 4b).

**Fig 5:**
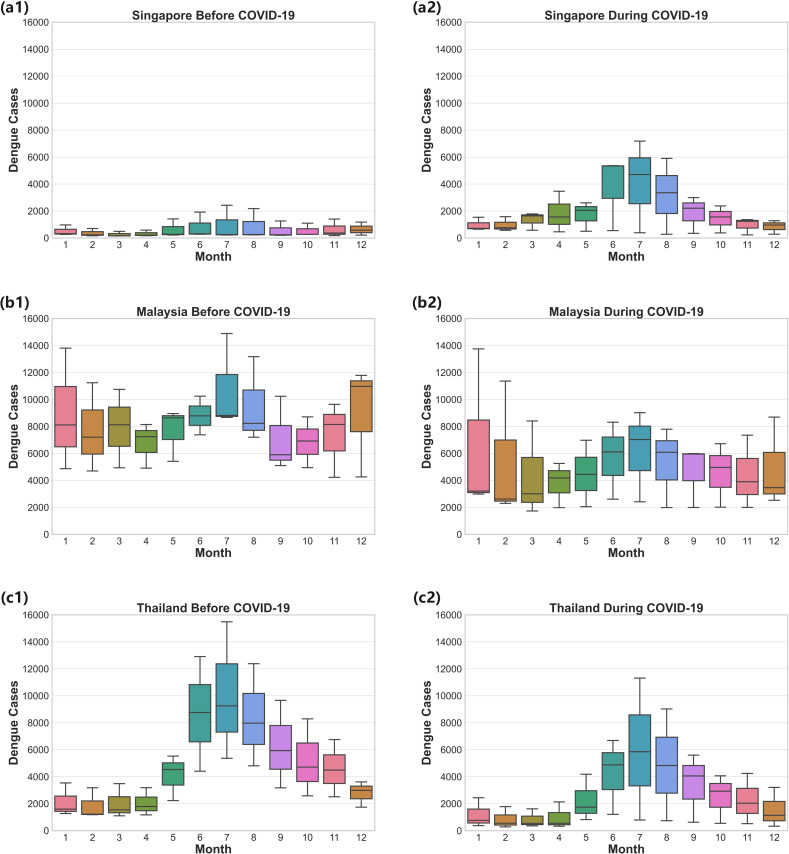
Box Plots of Seasonal Patterns of DF Incidences Before and After COVID- 19. a1. Singapore before COVID-19. a2. Singapore during COVID-19. b1. Malaysia before COVID-19. b2. Malaysia during COVID-19. c1. Thailand before COVID-19. c2. Thailand during COVID-19.

To better capture seasonality and explore the indirect impact of COVID-19, we use box plots to depict variations in each month’s DF cases, shown in Fig 5. Interesting patterns emerge across the three countries by comparing the seasonality plots before (2017–2019, shown by the left column) and during (2020–2022, shown by the right column) COVID-19. For Singapore, DF cases usually spiked sharply between May and August, which is consistent before and during the pandemic. However, the seasonality impact intensified with the presence of COVID-19: average monthly cases in the middle of a year increased from around 1,250–5,000, and the variability in infection cases across the same months increased markedly. For Malaysia and Thailand, a general reduction in DF cases throughout the year can be witnessed.

To investigate changes in the spatiotemporal cluster of DF before and during the COVID-19, we used retrospective spatiotemporal scanning statistical analysis. We found a high-risk cluster (RR = 11.84) was from April 2022 to September 2022 in Singapore (S6 Fig and S2 Table). Meanwhile, the low-risk cluster in Western Malaysia expanded and witnessed a reduction in RR (from 0.30 to 0.10) (S6 Fig and S1 and S2 Tables). Additionally, Thailand has consistently maintained a low risk, while a high-risk cluster (RR = 69.55) emerged in Mae Hong Son during DF season (S6 Fig and S2 Table).

### 3.3. Exposure-response relationship between environmental variables and DF incidence in pre-COVID-19 and during-COVID-19

We employed DLNM to unveil the exposure-response relationship between various environmental variables and DF, both pre-COVID-19 and during-COVID-19, across different study areas. Meanwhile, the overall cumulative association with a lag of 0–3 months was explored between environmental variables and DF cases in the same contexts.

**Fig 6:**
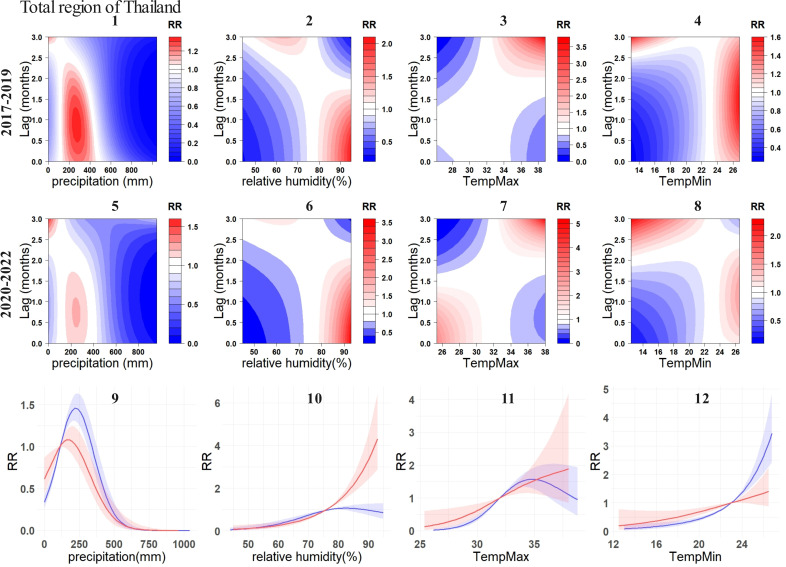
Exposure-response relationship between DF incidences and environmental variables in total region of Thailand. For subplots 1-4, the contour plots illustrate the relationship between four environmental variables and DF before the COVID-19, while subplots 5-8 depict these relationships during the COVID-19. 9-12 illustrate the overall cumulative associations (0-3 months) between the four environmental variables and DF. The blue lines represent the pre-COVID-19 (2017-2019), while the red lines represent the during-COVID-19 (2020-2022), with the corresponding 95% confidence intervals indicated in similar colors.

**Fig 7:**
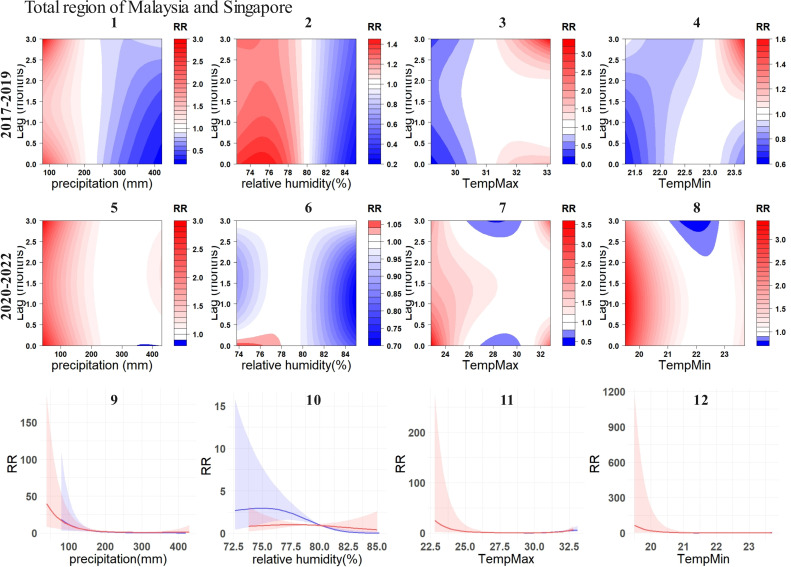
Exposure-response relationship between DF incidences and environmental variables in total region of Malaysia and Singapore. For subplots 1-4, the contour plots illustrate the relationship between four environmental variables and DF before the COVID-19, while subplots 5-8 depict these relationships during the COVID-19. 9-12 illustrate the overall cumulative associations (0-3 months) between the four environmental variables and DF. The blue lines represent the pre-COVID-19 (2017-2019), while the red lines represent the during-COVID-19 (2020-2022), with the corresponding 95% confidence intervals indicated in similar colors.

In a comprehensive overview, the overall exposure-response relationship between DF cases and environmental variables in total region of Thailand, as well as Malaysia and Singapore exhibited different changes (Figs 6 and 7). For Thailand, during the pandemic, the 0-1.5 months following the attainment of maximum temperatures between 25°C-30°C emerge as new occurrences of high-risk periods. In contrast, before pandemic, the RR within this maximum temperature range was exceedingly low (Fig 6c and 6g). For minimum temperature, the low RR interval was relatively broad before pandemic, occurring 0–3 months after the temperature reached around 20°C-23°C. During the COVID-19 period, areas previously characterized by low relative risk (RR) largely transitioned to high RR regions, with a maximum value of 3.3. It is noteworthy that this lagged relationship differs from the majority, as the RR sharply increases when the minimum temperature enters this interval, gradually decreasing over time, contrary to the typical pattern where RR tends to gradually rise with increasing lag duration (Fig 6d and 6h). Moreover, the overall cumulative association between environmental variables and RR in Thailand exhibited substantial changes with a lag of 0–3 months. Specifically, the peak of the overall cumulative association for precipitation and DF cases decreased by approximately 0.3 (Fig 6i). The association between relative humidity and DF cases underwent a significant transformation (Fig 6j). Pre-pandemic, the curve exhibited a slightly inverted U-shape, peaking at around 80%, with a value of approximately 1.0. However, during the COVID -19, the associations between relative humidity and RR became proportional, steadily increasing above around 80%, instead of decreasing. The maximum temperature curve has transitioned from an inverted U-shape to a positive correlation as temperatures reach their peak (Fig 6k). For minimum temperature, in comparison to the pre-pandemic period, a conspicuous decrease in RR has been observed when the minimum temperature exceeds 23 °C (Fig 6l).

Notably, in Malaysia and Singapore, apart from a general decline in relative risk (RR) associated with relative humidity, the other three environmental variables remained largely unchanged (Fig 7).

At the subregional scale in Thailand, the environmental variables with the exposure-response relationship to DF that varied considerably before and during the pandemic. Notable differences were observed for precipitation, relative humidity and maximum temperature in Northern Thailand, minimum temperature in Northeastern Thailand, maximum temperature in Eastern Thailand.

**Fig 8:**
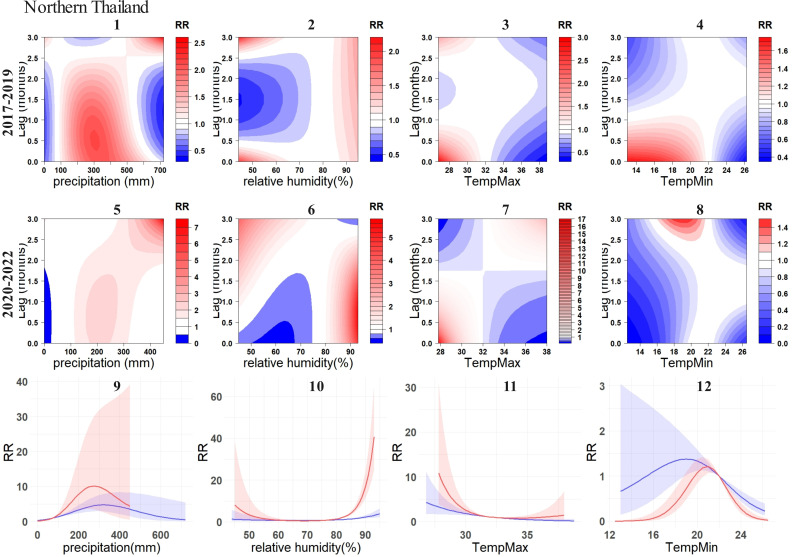
Exposure-response relationship between DF incidences and environmental variables in Northern Thailand. For subplots 1-4, the contour plots illustrate the relationship between four environmental variables and DF before the COVID-19, while subplots 5-8 depict these relationships during the COVID-19. 9-12 illustrate the overall cumulative associations (0-3 months) between the four environmental variables and DF. The blue lines represent the pre-COVID-19 (2017-2019), while the red lines represent the during-COVID-19 (2020-2022), with the corresponding 95% confidence intervals indicated in similar colors.

By contrast, the exposure-response relationship of environmental variables with DF cases were most variable in Northern Thailand (Fig 8). Specifically, high RR cluster of precipitation shows minimal variations in high RR areas. However, there is a significant elevation in RR values, with the highest value increasing from 2.5 to 7 (Fig 8a and 8e). The maximum temperature exhibited the most noticeable change among all environmental variables and regions (Fig 8c and 8g). Pre-pandemic, the high RR cluster was 0–1 month and 2.5-3 months after temperatures between 27°C-32°C. However, during the pandemic, there was a significant increase in RR within 0–1 month after this temperature range. Additionally, a new high RR zone emerged, 2.5-3 months after temperatures between 34°C-38°C. This suggests that the DF cases in Northern Thailand were highly influenced by the maximum temperature in these two temperature intervals during the pandemic. Interestingly, the previously identified high RR zone, 2.5-3 months after the pandemic, rapidly decreased to no risk, i.e., 0. For minimum temperature in the same region, the high RR zone also completely shifted its position (Fig 8d and 8h). Pre-pandemic, the high RR zone was 0–1 month after temperatures between 13°C-21°C. During the pandemic, a new high RR zone emerged, 2.5-3 months after temperatures between 17–22. The 0–3 months cumulative RR association between relative humidity and DF cases in Northern Thailand changed from a relatively flat curve to a clear U-shape, with a significant increase in RR (Fig 8j).

Northeastern Thailand’s minimum temperature high RR cluster completely shifted its position, demonstrating a highly significant change (S2d and S2h Fig). Pre-pandemic, the high RR interval occurred 0.5-1.5 months after the temperature reached 224°C-26°C, with the peak RR occurring one month after the lag. However, during the pandemic, this interval transitioned to a low-risk zone, and two new high RR zones emerged: 0-0.5 months and 2.5-3 months after the temperature reached 22°C-25°C. In Eastern Thailand (S4 Fig), there was a notable decline in RR associated with maximum temperature and DF incidence (S3c and S3g Fig), the maximum value has decreased from approximately 3.5°C to 1.5°C. Over a cumulative lag of 0–3 months, the peak RR for the association between precipitation and DF increased from approximately 1.8 to 3.4, with precipitation levels remaining constant at the peak (S3h Fig). Additionally, the peak value for relative humidity has decreased from about 3.3 to 1.1, accompanied by an approximate 2% increase in relative humidity when the peak occurs (S3i Fig). The exposure-response associations between DF incidences and environmental variables in Central Thailand and Southwestern Thailand changed little before and during COVID-19 (S4 and S5 Figs).

## 4. Discussion

### 4.1. Principal findings

In order to investigate the fluctuations in DF incidence during the pandemic, we employed time series analysis to examine the seasonality component of the monthly DF incidence data. We observed that three countries maintained pronounced seasonality in DF cases pre-and during-COVID-19. And a notable increase in DF cases in Singapore during COVID-19, especially from May to August, experiencing an approximately fourfold rise compared to the same period pre-COVID-19. Possibly attributed to increased human-mosquito contact opportunities due to population staying at home, a surge in blood supply [21]. In addition, vector control for DF primarily involves eliminating open water sources, insecticide spraying, and cleaning community drainage systems [1,55]. However, the COVID-19-related lockdowns and restrictions disrupted regular vector surveillance and control activities [15], which may have contributed to the surge in dengue cases observed in mid-2020. Studies have shown that high population density increases the risk of DF transmission [56,57]. During the COVID-19 pandemic, reduced human mobility, potentially resulting in localized increases in population density. This shift may have contributed to the surge in DF cases observed in mid-2020. And the southwestern monsoon (end of May to September) results in elevated temperatures, humidity, and precipitation levels conducive to mosquito proliferation [58]. And during COVID-19, both Thailand and Malaysia experienced a relatively significant decline in the DF cases. Possibly because Malaysia and Thailand potentially implemented divergent lockdown measures during the same period, while the timing and intensity of monsoons may have exhibited variations across the three countries. Furthermore, distinctions in healthcare infrastructure and the differences of public health interventions could contribute to disparate impacts on DF incidence in Singapore, Malaysia, and Thailand. Additionally, prior research has observed a slight delay in Thailand’s DF seasonality [28], but our study is the first to focus on the effect of intensified seasonality. While false positive DF test results in COVID-19 patients have been reported [25,26], this alone does not completely explain the observed seasonal variations. In 2020, the surge in DF cases coincided with Singapore’s COVID-19 lockdown period from April to June. However, in 2022, despite the easing of COVID-19 measures, DF incidences still surged in May. Moreover, as noted by Brady and Wilder-Smith [59], lockdowns are only temporary changes in human mobility and are unlikely to have a lasting impact on vector population or their behavior. Consequently, even in areas that saw a decrease in DF cases during lockdowns, it is possible that those infections were only postponed rather than prevented. Thus, other factors such as environment, socioeconomic status, and vector distribution should be examined over a more extended study period to establish a causal relationship between stay-at-home orders and DF surges. Through space-time scan statistics, it was found that during the pandemic, the overall RR in Thailand was relatively low, but Mae Hong Son became a high-risk area with an RR of 69.55. This was probably due to Mae Hong Son implemented sheltered sanitation practices and experienced the maximum temperature and miserable humidity levels, promoting the mosquito breeding [28].

The differences in the lagged relationship between environmental variables and DF cases before and during the pandemic reveal a more complex set of influencing factors. Previous research has indicated that an increase in temperature promotes the proliferation of Aedes aegypti and Aedes albopictus mosquitoes, enhancing the severity of DF infections [29]. Our study indicates that only the RR of DF cases in Northern Thailand showed a significant increase during the pandemic due to temperature variable. According to our data, the average monthly maximum temperature in Northern Thailand rose from 31.99 °C in 2017–2019 to 32.32°C in 2020–2022, marking an increase of 0.33 °C. In contrast, the differences in the monthly average maximum temperature in other parts of Thailand, as well as in Malaysia and Singapore, were extremely small, all below 0.05, with the differences in Southeast and Southwest Thailand, Malaysia, and Singapore even below 0.01. Therefore, the significantly soaring RR of the relationship between maximum temperature and DF in Northern Thailand may be attributed to the fact that the increase in maximum temperature, promoting breeding of Aedes aegypti and Aedes albopictus mosquitoes, and thus leads to an increase in DF infection. Additionally, concerning the exposure-response relationship between precipitation and the RR of DF, there was a significant increase in Northern Thailand during the pandemic. The high RR zone in this region was 2.5-3 months after extreme high rainfall (500mm-700mm) and during 0–2.5 months following the 100mm–500mm period before the pandemic. However, during the pandemic, this rainfall range shifted to around 350mm-400mm, with the lag time for high risk remaining roughly the same, resulting in a significant increase in RR, peaking at 7. This finding aligns with the observations by Cheng J et al. [35] in Guangzhou, China, indicating a significant increase in DF risk 6–13 weeks after extreme high rainfall. This observation is consistent with the perspective on vector, wherein mosquitoes exhibit heightened breeding frequency during the rainy season [21]. Apart from Northern Thailand, variations in precipitation were observed in other study regions, but the changes in RR were generally small. Therefore, the exposure-response relationship between precipitation and DF RR exhibits regional differences, necessitating further investigation.

Notably, the overall cumulative associations between relative humidity and DF incidence in Thailand reveal an inverted U-shaped relationship, peaking at approximately 70%–75% relative humidity across all regions except the North. This pattern aligns with previous research findings [24]. Additionally, the COVID-19-related restrictive measures reduced overall human mobility [60,61], which likely decreased human-mosquito contact in the relative humidity range most conducive to DF transmission. This reduction may explain the observed decline in relative risk (RR) of DF infection during non-sensitive periods (i.e., relative humidity below 70%–75%), particularly in regions outside of northern Thailand, such as the Southeast, East, Central, and Southwest (S1, S3, S4 and S5 Figs). Previous studies have indicated that COVID-19 had limited impact on agricultural and industrial labor forces [62]. As a result, during the DF-sensitive summer season, when agricultural activities peak and relative humidity is high, human-mosquito contact increased. This may account for the relatively stable RR of DF infection during the two periods in all regions except the North, with notable consistency in the East and Central regions (S3 and S4 Figs).

### 4.2. Implications and recommendations

The COVID-19 pandemic has posed one of the most significant public health challenges. Despite the low likelihood of a similar pandemic in the near future, the insights gained from this unique situation regarding DF transmission can contribute to our knowledge gap and be applied in future DF control studies [4].

First and foremost, we emphasize the critical importance of vector control in preventing DF outbreaks. Our study reveals the region-specific effects of pandemic-related measures on DF outbreaks. Although measures such as lockdowns can potentially reduce DF transmission by limiting mobility and socio-economic activities, it is also likely that residents staying at home increase the risk of mosquito breeding in residential areas [21], as seen in Singapore. Therefore, future DF control efforts should prioritize vector control and the prevention of vector breeding [63,64].

Secondly, we urge relevant authorities to strategically allocate their healthcare resources to ensure proper DF management even during times of emergency. The allocation of healthcare resources has been put to the test during this public health crisis. Many countries struggled to cope with the sudden pressure on limited resources, resulting in neglect of the DF situation with discontinued vector control and inadequate DF testing [64–66]. Relevant authorities can proactively intervene based on the environmental conditions associated with an elevated DF infection rate. For instance, in areas experiencing increased precipitation, implementing artificial rainfall reduction measures may be a strategic intervention to reduce mosquito breeding rates [67]. Additionally, targeted weed control measures could be employed to minimize breeding zones. Moreover, distributing mosquito repellent and nets [28] to residents can help protect them from mosquitoes even if vector control measures are not effective.

Thirdly, we urge SEA countries to strengthen their collaborative efforts in combatting DF due to DF transmission mechanisms is complex and country-specific. Localized clusters were prevalent during the pandemic. However, as the world enters the post-COVID recovery era, which sees increasing mobility both within and across country borders, cross-country clusters are increasing. This situation may be exacerbated by the regional synchrony of DF risk, highlighting the need for regional collaboration in DF control [68]. The SEA countries should share relevant data and the latest findings with each other as soon as possible, promoting co-operation and minimizing the risk [69,70].

Last but not least, the exposure-response relationships between environmental variables and DF incidence shifted between 2017–2019 (pre-COVID-19) and 2020–2022 (during-COVID-19) across different regions. These changes may be due to the effects of COVID-19 lockdowns or other natural factors [71,72], altering DF transmission dynamics [34,35]. We recommend that health authorities monitor the rise in DF infections linked to shifts in human mobility or short-term environmental changes and implement early preventive measures. However, the relationship between environmental factors and DF is complex and influenced by multiple variables [2]. Further research is needed to better understand these interactions.

### 4.3. Limitations

Despite gaining valuable insights, our results have been limited by various obstacles encountered during the studies on DF transmission. A significant challenge has been the limited availability of DF incidence data, even among the countries that do make their data available, many only provide it at a coarse spatial scale, such as the national level, which limits the possibility of uncovering finer details. Therefore, this study was only able to analyze regional trends in SEA using data from three countries that were available at a suitable spatial scale.

Moreover, variations in DF cases during the pandemic are not simply due to under-reporting of data for healthcare stress caused by COVID-19. [4,14] Therefore, it is important to conduct further studies to explore the possibility and extent of under-reporting and other reasons of cases reduction during pandemics to minimize data uncertainty and draw significant conclusions about the indirect impact of pandemics on DF transmission.

Lastly, the global response to COVID-19 measures has led to a reduction in nitrogen oxide emissions, thereby promoting short-term warming [73], which could potentially influence the DF incidences. However, this study did not confirm that the differences in the exposure-response relationships between environmental variables and DF incidences before and during the pandemic were definitely influenced by COVID-19. Instead, it is plausible that various environmental factors underwent short-term and slight changes for different reasons during these two periods. Further investigation is required to ascertain what factors are statistically significantly related to the changes of exposure-response relationship between environmental variables and DF over the two time periods.

## 5. Conclusion

By using time series analysis, we improve our understanding of indirect impact of COVID-19 on DF transmission in each of the three countries studied. During the COVID-19 pandemic, DF transmission patterns shifted across the three studied countries. Singapore saw a significant surge in cases during 2020 and 2022, while Thailand exhibited strong synchrony in DF trends across subregions, except in 2021. Finally, by employing a distributed lag nonlinear model with NPI, we identified varying degrees of spatial heterogeneity in the exposure-response relationships between environmental variables and DF incidences both pre- and during-COVID-19, particularly in northern Thailand. Our findings highlight that the changes in human mobility patterns resulting from government interventions against COVID-19, coupled with the indirect impacts on short-term environmental change, can exert varying degrees of DF transmission.

## Supporting information

S1 Fig Exposure-response relationship and overall cumulative associations between dengue incidences and climate variables in Southeastern Thailand. (TIF)

S2 Fig Exposure-response relationship and overall cumulative associations between dengue incidences and climate variables in Northeastern Thailand. (TIF)

S3 Fig Exposure-response relationship and overall cumulative associations between dengue incidences and climate variables in Eastern Thailand. (TIF)

S4 Fig Exposure-response relationship and overall cumulative associations between dengue incidences and climate variables in Central Thailand. (TIF)

S5 Fig Exposure-response relationship and overall cumulative associations between dengue incidences and climate variables in Southwestern Thailand. (TIF)

S1 Text Methods, results and references of Retrospective Poisson Space-Time Scan Statistic. The results include S6 Fig and S1 and S2 Tables.
(DOCX)

S6 Fig Spatial distribution of space-time clusters of dengue fever cases in 2017–2019 and 2020–2022. (The base layer of the map is from GADM database of Global Administrative Areas, Version 4.1, with license information at https://gadm.org/data.html).
(TIF)

S1 Table Space-time clusters of dengue incidences pre-COVID-19 (2017–2019). (DOCX)

S2 Table Space-time clusters of dengue incidences during-COVID-19 (2020–2022). (DOCX)
