# Supplementary figures and images for: Unraveling varying spatiotemporal patterns of Dengue Fever and associated exposure-response relationships with environmental variables in three Southeast Asian countries before and during COVID-19
Source: PLoS Negl Trop Dis. 2025 Apr 28;19(4):e0012096. doi: 10.1371/journal.pntd.0012096 (PMC12121919; doi:10.1371/journal.pntd.0012096)

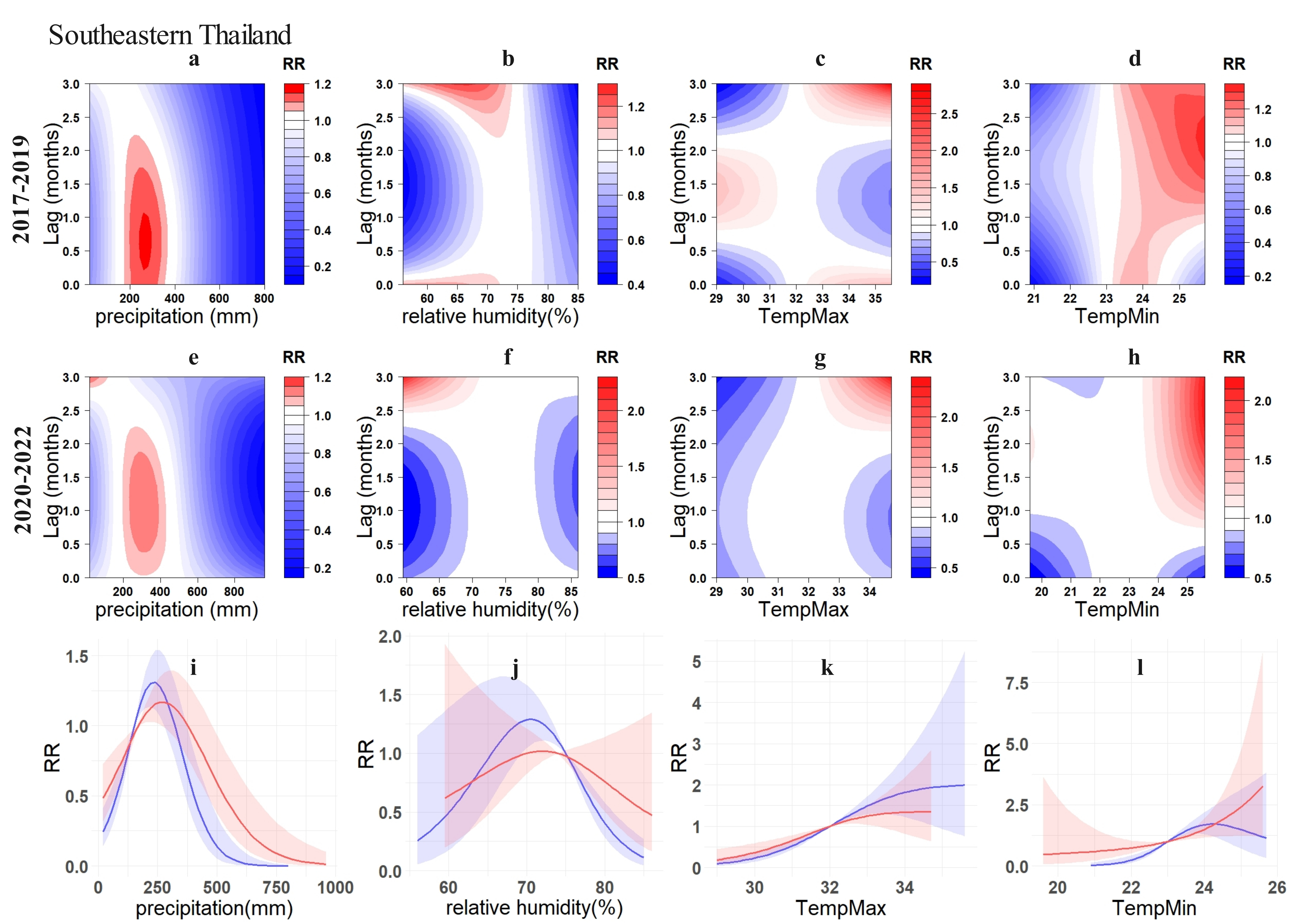

Supplement: S1 Fig — (TIF) [file pntd.0012096.s001.tif]

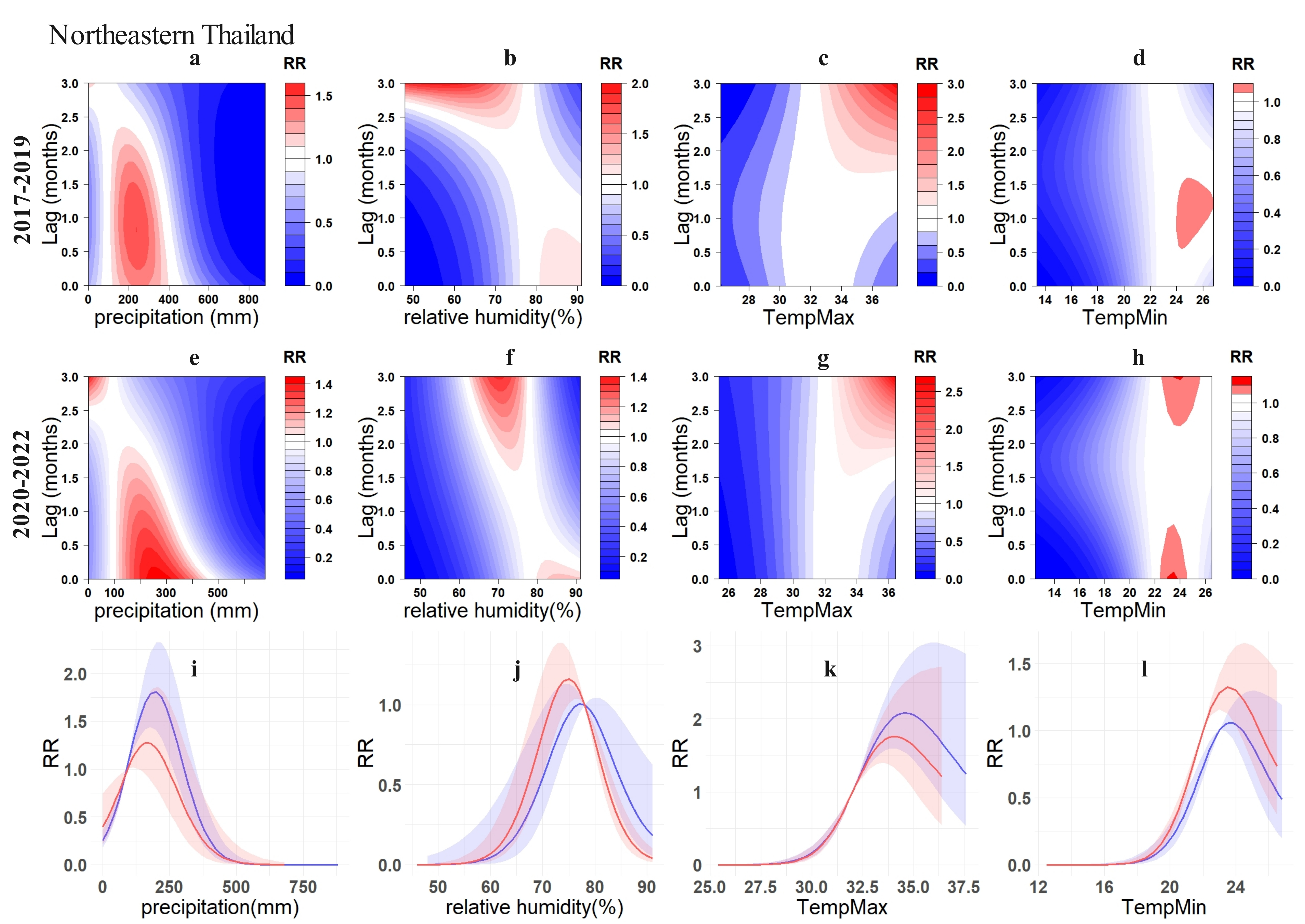

Supplement: S2 Fig — (TIF) [file pntd.0012096.s002.tif]

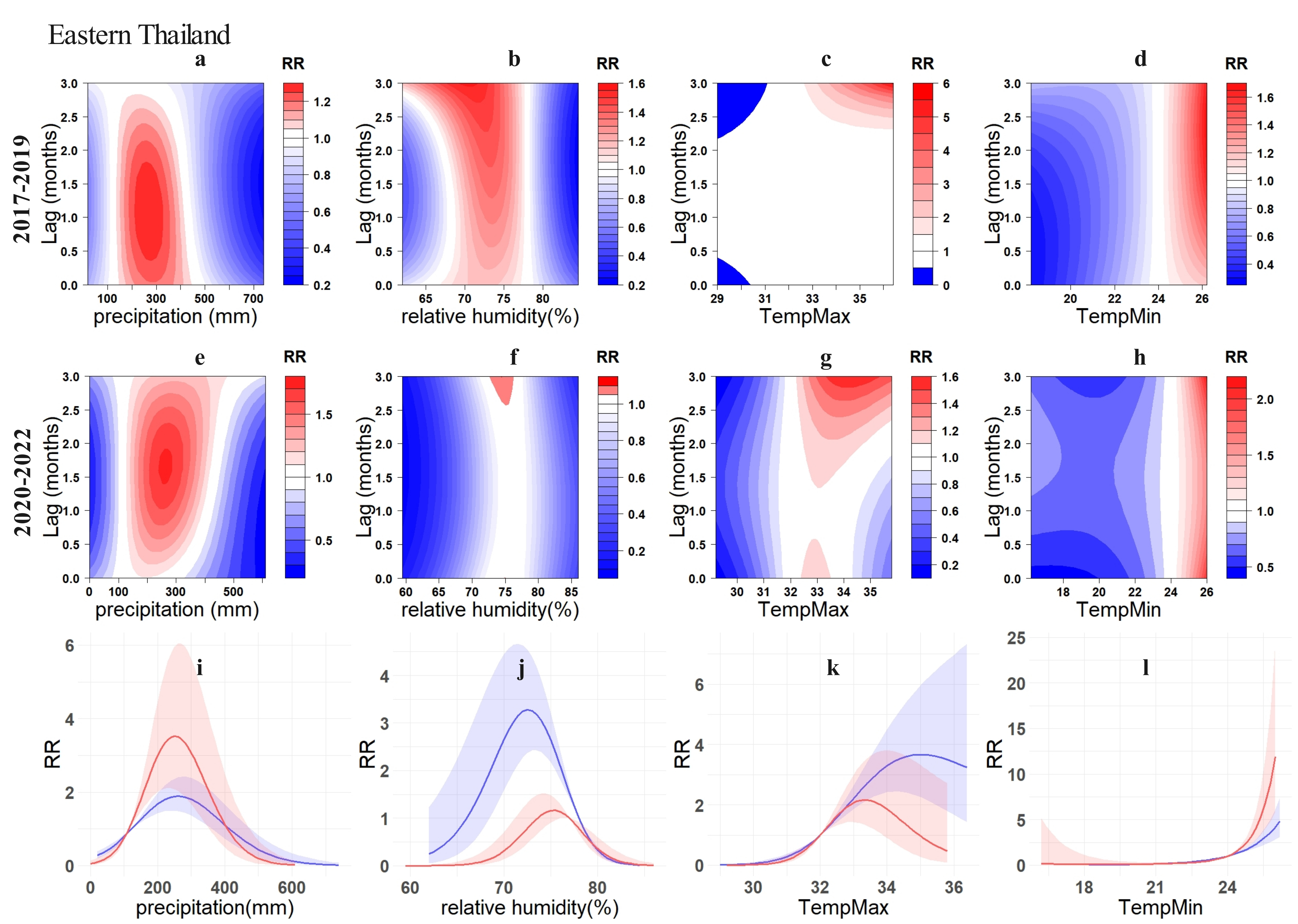

Supplement: S3 Fig — (TIF) [file pntd.0012096.s003.tif]

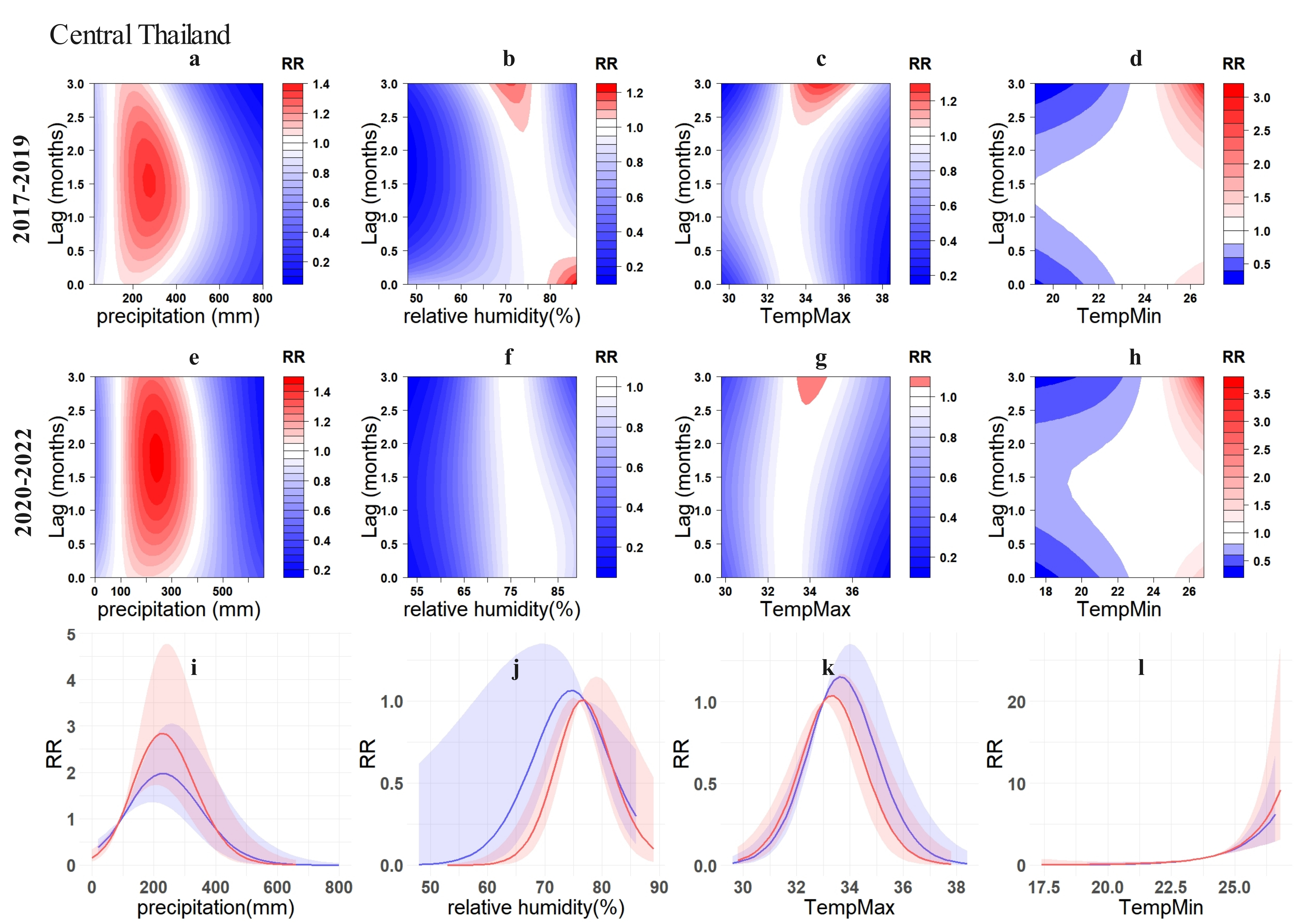

Supplement: S4 Fig — (TIF) [file pntd.0012096.s004.tif]

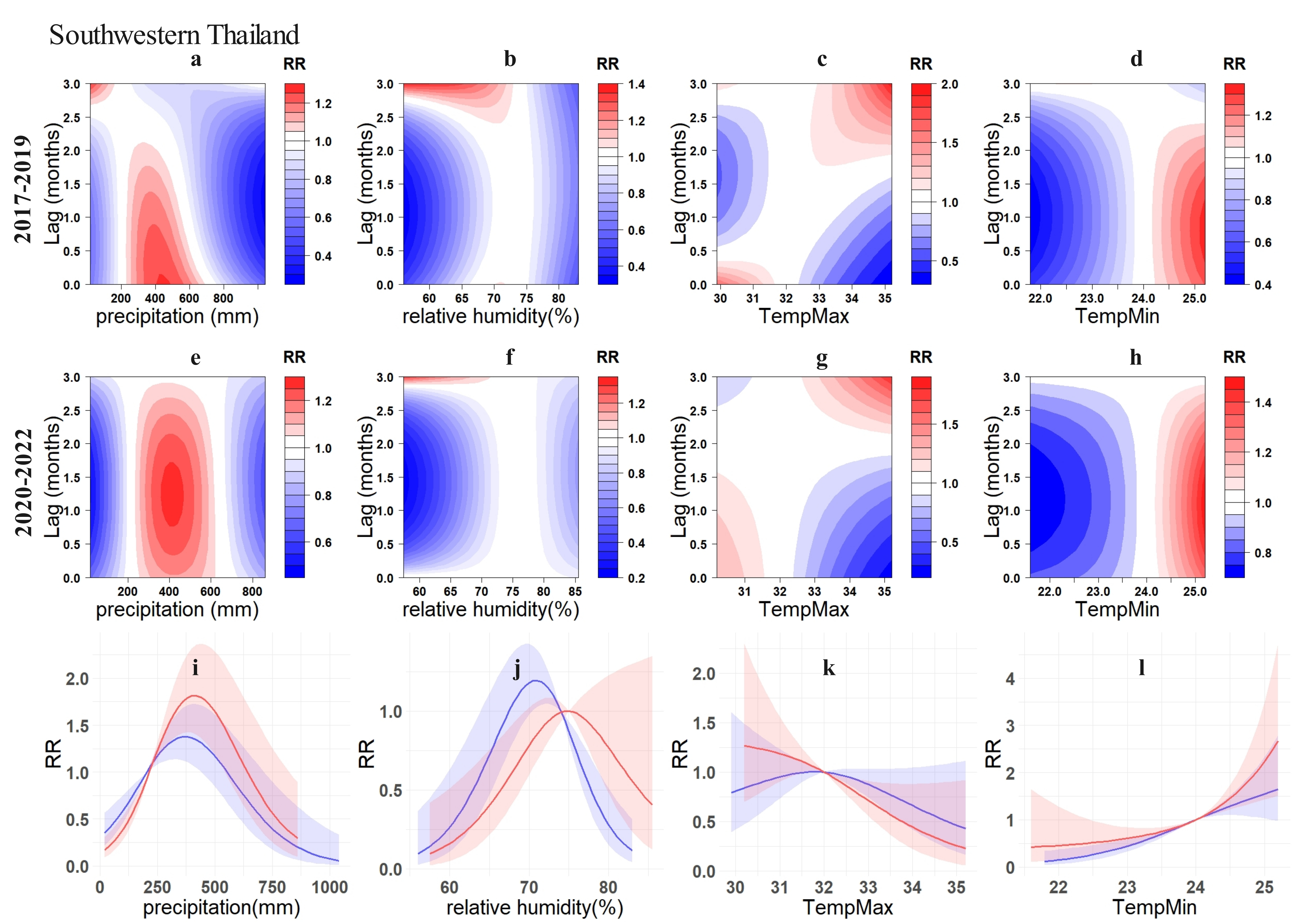

Supplement: S5 Fig — (TIF) [file pntd.0012096.s005.tif]

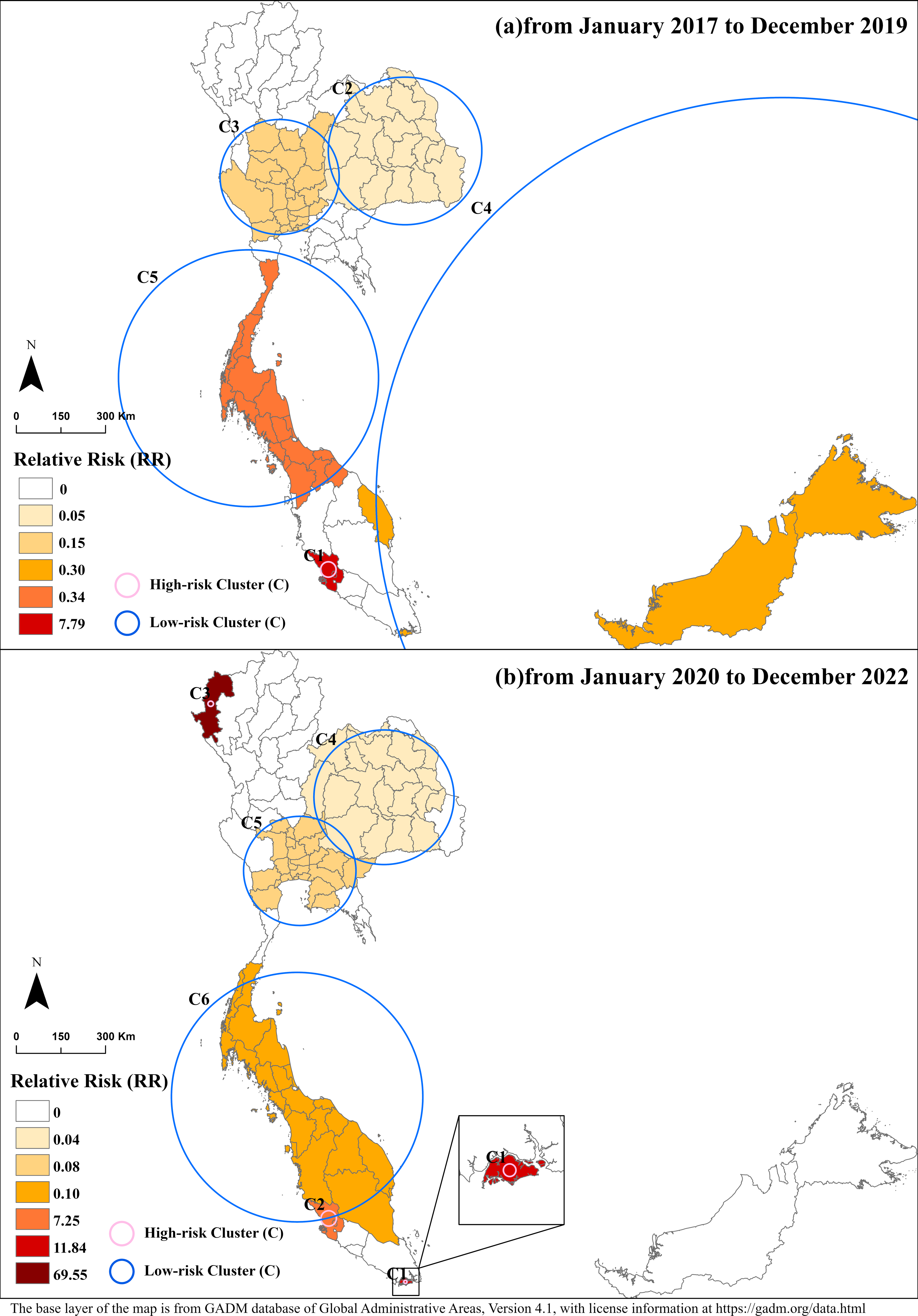

Supplement: S6 Fig — (The base layer of the map is from GADM database of Global Administrative Areas, Version 4.1, with license information at https://gadm.org/data.html). (TIF) [file pntd.0012096.s007.tif]
