# Supplementary material for: Unraveling varying spatiotemporal patterns of Dengue Fever and associated exposure-response relationships with environmental variables in three Southeast Asian countries before and during COVID-19
Source: PLoS Negl Trop Dis. 2025 Apr 28;19(4):e0012096. doi: 10.1371/journal.pntd.0012096 (PMC12121919; doi:10.1371/journal.pntd.0012096)
Supplement: S1 Text — The results include S6 Fig and S1 and S2 Tables. (DOCX) [file pntd.0012096.s006.docx]

1. **Retrospective Poisson Space-Time Scan Statistic**

1.1 Method of Space-Time Scan Statistic

Spatio-temporal analysis is a common method for investigating dengue transmission patterns [[1–4], so this study compares patterns before and during the pandemic to offer valuable insights](#bookmark26) into the impact of COVID-19 on dengue. Space-time scan statistics is widely used for exploring spatio-temporal clusters [[5–7], and we adopt this method to identify high- and low-risk clusters](#bookmark27) and delineate the pandemic’s role in dengue’s spatial variations. Hence, we employ retrospective Space-Time Scan Statistical analysis using SaTScan v10.1 [8] [for the identification of space-time](#bookmark28) clusters of dengue fever cases during the study period. This method allows the detection of statistically significant clusters, both high- and low-risk ones, with the consideration of uneven population distribution.

This method assumes that the number of dengue fever cases follows a Poisson distribution. A cylindrical window is adopted for the detection of clusters, with its height and base representing time and space, respectively. The center of the cylinder represents the centroids of the area under study. The window used in this study is expanded in both space and time until it reaches pre-defined maximum boundaries of 20% of the population at risk and 30% of the study period, respectively. After experimenting with different configurations, these boundaries were chosen. The spatial boundary prevents the identification of localized clusters that consist of only one province, making it easier to analyze regional synchrony. Meanwhile, the temporal boundary ensures that the cluster identified when dividing the study period into 3-year intervals does not exceed a year. This approach allows for a more precise capture of dengue seasonality.

For each possible space-time cluster, hypothesis testing is performed, where the null hypothesis (H0) indicates the intensity of dengue fever is the same within and outside the cylinder. The alternative hypothesis (HA) indicates the number of observed cases exceeds the expected ones, highlighting higher risk within the cylinder. Expected cases are computed by Equation [1,](#bookmark29) where μ denotes expected cases, p denotes the population of the unit under study, and C and P represent the total number of dengue cases observed in and total population of 3 SEA countries, respectively.

The statistic used for hypothesis testing is defined by Equation [2,](#bookmark30) where L(Z) and L0 denote the likelihood function for cylinder Z and H0, respectively, cz denotes the number of cases in cylinder Z, and μz represent the expected cases within Z.

$\begin{aligned} \mu=p\times\frac{C}{P}\#\left( S1 \right) \end{aligned}$

$$\begin{aligned} \frac{L\left( Z \right)}{L_{0}}=\frac{\left( \frac{c_{Z}}{\mu\left( Z \right)} \right)^{c_{Z}}\left( \frac{C-c_{Z}}{C-\mu\left( Z \right)} \right)^{C-c_{Z}}}{\left( \frac{C}{\mu\left( T \right)} \right)^{C}}\#\left( S2 \right) \end{aligned}$$

To quantify the risk level of each cluster and ease comparison across clusters, Relative Risk (RR) is defined, which is captured by Equation [3.](#bookmark31) c and μ represent the number of actual dengue cases and expected cases in the cluster, respectively.

$$\begin{aligned} RR=\frac{c/\mu}{\left( C-c \right)/\left( C-\mu\right)}\#\left( S3 \right) \end{aligned}$$

High-risk clusters have RR > 1, indicating the risk of suffering from dengue cases is higher than expected. The opposite applies to low-risk clusters. For a cluster with an RR value of 2.5, people living in the cluster are 2.5 times more likely to have dengue fever.

We divide the study period into two parts, the pre-COVID period (2017-2019) and the during- COVID period (2020-2022). For each period, retrospective space-time scans are performed to identify all clusters that have ever existed within the study period.

**References**

1. Aswi A, Cramb SM, Moraga P, Mengersen K. Bayesian spatial and spatio-temporal approaches to modelling dengue fever: a systematic review. Epidemiology & Infection. 2019 Jan;147:e33.

2. Lowe R, Bailey TC, Stephenson DB, Graham RJ, Coelho CA, Carvalho MS, Barcellos C. Spatio-temporal modelling of climate-sensitive disease risk: Towards an early warning system for dengue in Brazil. Computers & Geosciences. 2011 Mar 1;37(3):371-81.

3. Jeefoo P, Tripathi NK, Souris M. Spatio-temporal diffusion pattern and hotspot detection of dengue in Chachoengsao province, Thailand. International journal of environmental research and public health. 2011 Jan;8(1):51-74.

4. Estallo EL, Carbajo AE, Grech MG, Frías-Céspedes M, López L, Lanfri MA, Ludueña-Almeida FF, Almiron WR. Spatio-temporal dynamics of dengue 2009 outbreak in Córdoba City, Argentina. Acta tropica. 2014 Aug 1;136:129-36.

5. Nisha V, Gad SS, Selvapandian D, Suganya V, Rajagopal V, Suganti P, Balraj V, Devasundaram J. Geographical information system (GIS) in investigation of an outbreak. The Journal of communicable diseases. 2005 Mar 1;37(1):39-43.

6. Schmidt WP, Suzuki M, Dinh Thiem V, White RG, Tsuzuki A, Yoshida LM, Yanai H, Haque U, Huu Tho L, Anh DD, Ariyoshi K. Population density, water supply, and the risk of dengue fever in Vietnam: cohort study and spatial analysis. PLoS medicine. 2011 Aug 30;8(8):e1001082.

7. Liu C, Liu Q, Lin H, Xin B, Nie J. Spatial analysis of dengue fever in Guangdong Province, China, 2001-2006. Asia Pacific Journal of Public Health. 2014 Jan;26(1):58-66.

8. Kulldorff M, Rand K, Gherman G, Williams G, DeFrancesco D. SaTScan–software for the spatial and space–time scan statistics, version 2.1. Bethesda, Madison: National Cancer Institute. 1998.
