# Supplementary material for: Unraveling varying spatiotemporal patterns of Dengue Fever and associated exposure-response relationships with environmental variables in three Southeast Asian countries before and during COVID-19
Source: PLoS Negl Trop Dis. 2025 Apr 28;19(4):e0012096. doi: 10.1371/journal.pntd.0012096 (PMC12121919; doi:10.1371/journal.pntd.0012096)
Supplement: S1 Table — (DOCX) [file pntd.0012096.s008.docx]

**Table S1.** Space-time clusters of dengue incidences pre-COVID-19 (2017 to 2019)

| Cluster | Duration | Total provinces, N | P value | Observed | Expected | Relative Risk (RR) |
| --- | --- | --- | --- | --- | --- | --- |
| 1 | Jun. 2019 -Dec. 2019 | 2 | <.001 | 51,395 | 7,300.92 | 7.79 |
| 2 | Oct. 2017-Apr. 2018 | 18 | <.001 | 986 | 18035.04 | 0.05 |
| 3 | Jan. 2017 - Jun. 2017 | 22 | <.001 | 2,368 | 15,215.30 | 0.15 |
| 4 | Mar. 2017 - Sep. 2017 | 5 | <.001 | 3,656 | 11901.05 | 0.30 |
| 5 | Oct. 2017 - Apr. 2018 | 17 | <.001 | 3,739 | 10,726.66 | 0.34 |
