# Supplementary material for: Unraveling varying spatiotemporal patterns of Dengue Fever and associated exposure-response relationships with environmental variables in three Southeast Asian countries before and during COVID-19
Source: PLoS Negl Trop Dis. 2025 Apr 28;19(4):e0012096. doi: 10.1371/journal.pntd.0012096 (PMC12121919; doi:10.1371/journal.pntd.0012096)
Supplement: S2 Table — (DOCX) [file pntd.0012096.s009.docx]

**Table S2.** Space-time clusters of dengue incidences during-COVID-19 (2020-2022)

| Cluster | Duration | Total provinces, N | P value | Observed | Expected | Relative Risk (RR) |
| --- | --- | --- | --- | --- | --- | --- |
| 1 | Apr. 2022 - Sep. 2022 | 1 | <.001 | 22940 | 2,072.05 | 11.84 |
| 2 | Jan. 2020 - Jul. 2020 | 2 | <.001 | 39,674 | 6,115.37 | 7.25 |
| 3 | May 2022 - Sep. 2022 | 1 | <.001 | 5,120 | 74.78 | 69.55 |
| 4 | Nov. 2020 - May. 2021 | 19 | <.001 | 632 | 14,960.09 | 0.04 |
| 5 | Apr. 2021 - Oct. 2021 | 22 | <.001 | 1,305 | 15,171.17 | 0.08 |
| 6 | Jan. 2021 - Jul. 2021 | 21 | <.001 | 1,626 | 15,228.66 | 0.10 |
